# Surface Hydrogenation Strategy for Constructing a 2D B_12_X_2_H_8_ (X = N, P, As) Family with Embedded Aromatic Icosahedral B_12_ Superatoms

**DOI:** 10.3390/nano16181127

**Published:** 2026-09-09

**Authors:** Lu-Yao Tian, Hao-Ning Li, Jun-Hui Yuan, Pan Zhang, Jiafu Wang

**Affiliations:** 1 School of Integrated Circuits, Peking University, Beijing 100871, China; 2 School of Biomedical Engineering, South-Central Minzu University, Wuhan 430074, China; 3 School of Physics and Mechanics, Wuhan University of Technology, Wuhan 430070, China

**Keywords:** two-dimensional materials, B_12_ superatom, semiconductor, carrier mobility, first-principles calculations

## Abstract

Surface hydrogenation is a key strategy for material modification. The icosahedral B_12_ superatom, featuring a closed-shell electronic structure and aromatic stability, serves as an ideal building block for 2D functional materials. Using first-principles calculations, we design highly stable 2D boron-based *h*-B_12_X_2_H_8_ (X = N, P, As) by hydrogenating the parent *h*-B_12_X_2_ phases proposed in our previous work. Hydrogenation widens the bandgap from ~1 eV to 5.19–6.00 eV, strengthens bonding, and improves mechanical properties (higher Young’s modulus and lower Poisson’s ratio). Modified deformation-potential theory reveals carrier-type-selective mobilities, with the electron mobility of *h*-B_12_P_2_H_8_ reaching 1755 cm^2^V^−1^s^−1^. Notably, when *h*-B_12_X_2_H_8_ forms a heterojunction with its parent phase, it acts as a protective layer that preserves the parent’s electronic structure, facilitating applications in harsh environments. This work provides a rational pathway for designing B_12_-based 2D materials via surface passivation and offers a model for constructing self-passivating protective layers on 2D materials.

## 1. Introduction

Since the successful exfoliation of graphene, two-dimensional (2D) materials have attracted considerable attention due to their unique electronic, optical, and mechanical properties [1,2]. Systems such as transition metal dichalcogenides (TMDs) hold great promise for optoelectronic applications [3]; however, intrinsic defects and environmental instability (e.g., the facile oxidation of phosphorene and borophene in air) severely impede their practical deployment [4,5,6]. Thus, how to simultaneously improve structural stability and environmental robustness has become a core challenge in this field.

Surface passivation, as an efficient post-treatment modification approach, can eliminate surface dangling bonds and defect states while precisely modulating the band structure without altering the bulk phase [7,8,9]. Previous studies have shown that chemical passivation can significantly enhance the photoluminescence efficiency and charge transport performance of monolayer TMDs [10,11], and ligand-mediated passivation can also effectively suppress the oxidative degradation of MXenes [12,13]. Among various passivation techniques, surface hydrogenation has garnered particular interest due to its simplicity, high effectiveness, and reversible tunability [13,14]. Both theoretical calculations and experiments have confirmed that hydrogenation markedly strengthens the structural stability of borophene: the oxidation rate of hydrogenated borophene in air is reduced by more than two orders of magnitude, and the intrinsic properties can be fully restored through dehydrogenation upon thermal annealing [15,16,17,18]. Moreover, hydrogenation enables targeted modulation of electronic properties, as verified in systems such as graphene and arsenene [19,20,21,22]. For example, hydrogenation has been shown to simultaneously enhance the structural stability and superconducting performance of β_12_ borophene [23], highlighting the unique advantages of surface hydrogenation in precisely tailoring the properties of boron-based 2D materials.

Based on the above research progress and existing challenges, this work proposes a new strategy for constructing 2D materials via surface hydrogenation, using our previously theoretically proposed *h*-B_12_X_2_ structures [24] as templates to construct a series of 2D materials, *h*-B_12_X_2_H_8_ (X = N, P, As). We systematically investigate their lattice configurations, electronic band structures, in-plane mechanical parameters (Young’s modulus and Poisson’s ratio), and carrier mobilities and further explore the electronic properties when they form heterojunctions with the parent *h*-B_12_X_2_ materials, thereby providing a comprehensive theoretical foundation for understanding *h*-B_12_X_2_H_8_.

## 2. Methods

All calculations were performed using the VASP code [25,26], employing the projector augmented-wave (PAW) method [27,28] within the generalized gradient approximation (GGA) [29]. The kinetic energy cutoff of plane-wave basis set was set to 500 eV. The exchange-correlation energy was expressed using the Perdew-Burke-Ernzerhof (PBE) functional [29]. The valence electron configurations were set as follows: 1*s*^1^ for H, 2*s*^2^2*p*^1^ for B, 2*s*^2^2*p*^3^ for N, 3*s*^2^3*p*^3^ for P, and 4*s*^2^4*p*^3^ for As. For structural optimization, a strict force criterion of 0.005 eV/Å in all directions was employed, in addition to a dense *k*-mesh (9 × 9 × 1). Furthermore, a vacuum spacing of 15 Å along the *z* direction was chosen to ensure that the interlayer separation is sufficiently large to converge the total energy and electrostatic potential, guaranteeing that the calculated electronic and structural properties are intrinsic to an isolated monolayer, free from any interference from adjacent periodic replicas. The thermal stability of the 2D monolayers was evaluated through ab initio molecular dynamics (AIMD) simulations. These were carried out at various temperatures for 5 ps using a 3 × 3 × 1 supercell for 2D monolayers. Phonon dispersion calculations were performed using PHONOPY software [30]. To overcome the underestimation of bandgaps in semiconductors or insulators by the GGA-PBE method [31,32,33,34], we introduced the hybrid functional HSE06 [35] to evaluate the bandgaps. Additionally, the intermolecular interactions were described using Grimme’s DFT-D3 dispersion correction [36]. The VASPKIT [37] and VESTA [38] software programs were employed in the process of data analysis.

## 3. Results

**Figure 1:**
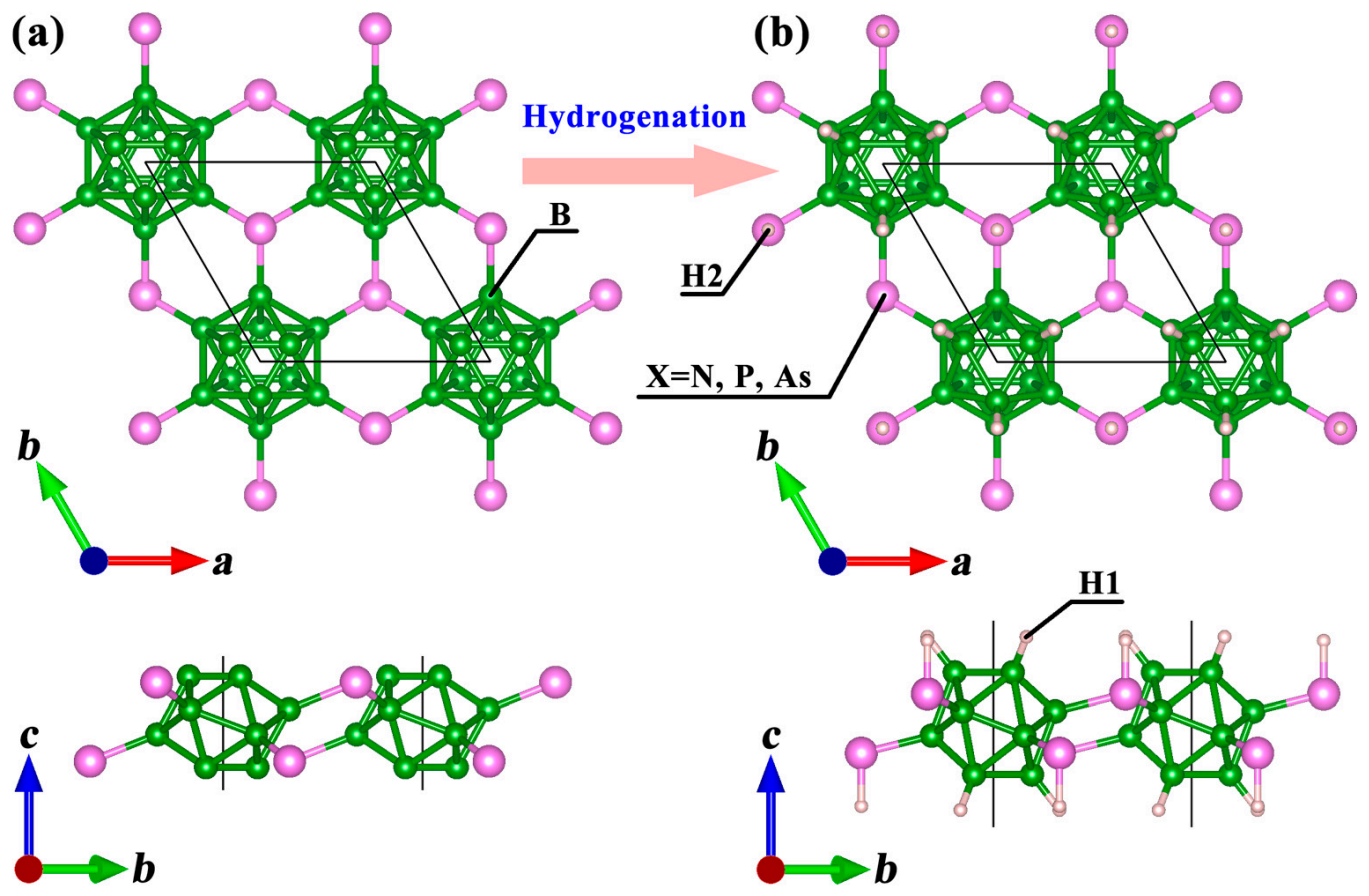
Design concepts of 2D *h*-B_12_X_2_H_8_ (X = N, P, As). Top and side views of (**a**) *h*-B_12_X_2_ and (**b**) *h*-B_12_X_2_H_8_ (X = N, P, As). To distinguish the two inequivalent hydrogen sites in *h*-B_12_X_2_H_8_, we labeled them as H1 (bonded to B) and H2 (bonded to X), respectively.

In our previous work, we predicted monolayer *h*-B_12_X_2_ (X = N, P, As) by exfoliating bulk boride parent phases based on first-principles calculations [24], with the structure shown in Figure 1a. Previous studies revealed that *h*-B_12_X_2_ are a class of narrow-bandgap semiconductors with excellent carrier transport properties. However, this material system suffers from surface stability issues: both B and X sites exhibit strong adsorptivity and are susceptible to environmental influences. To address this issue, we propose to modify *h*-B_12_X_2_ via surface hydrogenation, which has been proven to be an effective approach in numerous studies [39,40]. The design scheme is illustrated in Figure 1b. The hydrogenation strategy is based on two considerations: first, the hydrogenation of the B_12_ superatom surface echoes the structural chemistry of borane ($B_{12}H_{12}^{2-}$); second, the X sites (group VA elements) intrinsically possess lone-pair electrons that can be utilized to form saturated passivation bonds. On this basis, we initially constructed the fully hydrogenated product *h*-B_12_X_2_H_8_, whose structure is also shown in Figure 1b.

Subsequently, we performed high-precision geometry optimization on the designed structures and extracted the lattice parameters, which are listed in Table 1 along with the corresponding data of the parent *h*-B_12_X_2_ for comparison. The results show that the lattice constants of *h*-B_12_X_2_H_8_ are slightly reduced compared to the parent phases, with differences of only 0.7–1.4%, indicating that hydrogenation has a negligible effect on the overall lattice dimensions. Moreover, the B–B bond lengths within the B_12_ superatom become more uniformly distributed after hydrogenation, while the changes in B–X bond lengths are also very small. These results demonstrate that hydrogenation has only a limited impact on the overall structure of *h*-B_12_X_2_, effectively ensuring structural consistency and continuity before and after hydrogenation.

**Figure 2:**
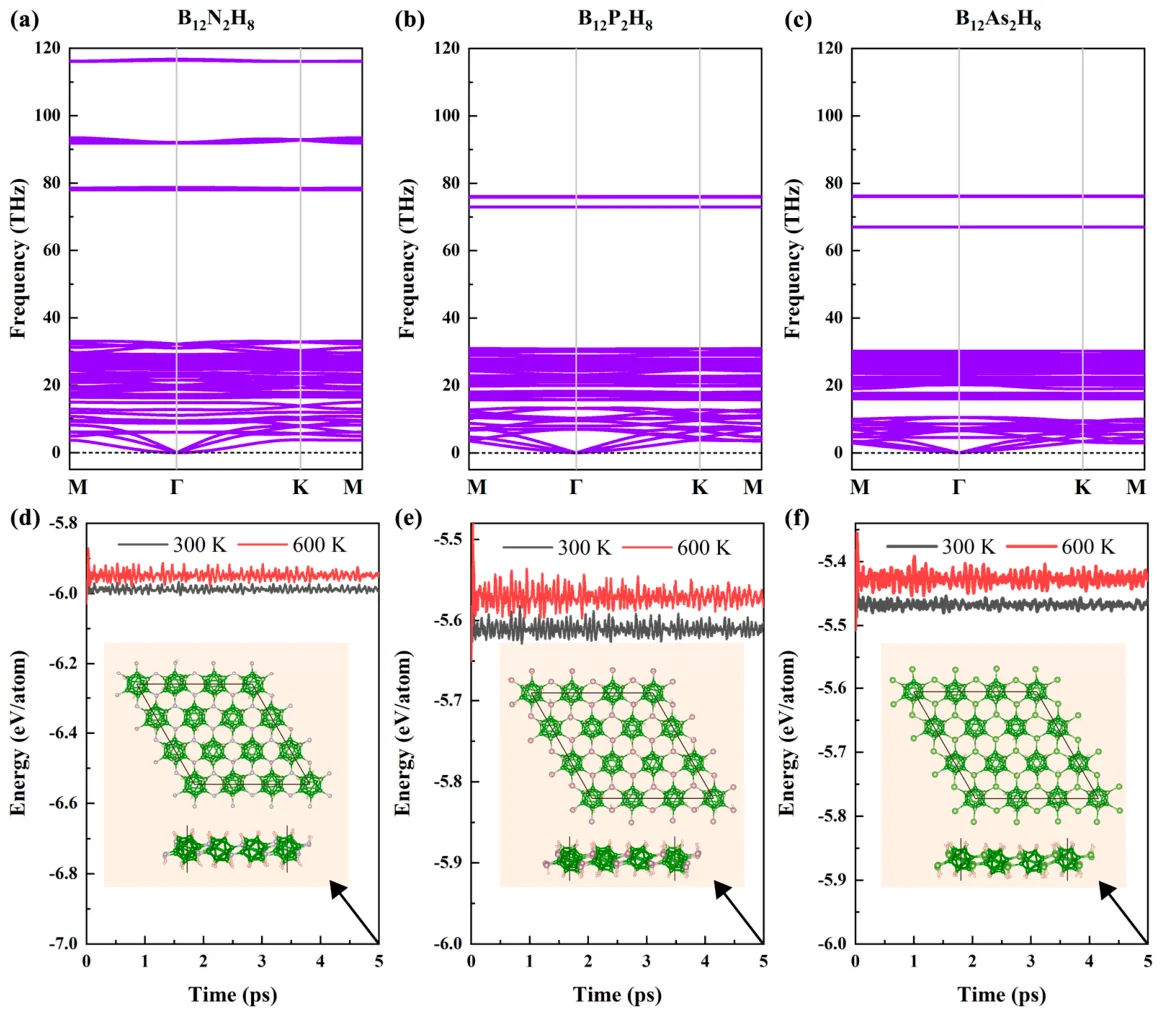
Phonon dispersions of (**a**) *h*-B_12_N_2_H_8_, (**b**) *h*-B_12_P_2_H_8_ and (**c**) *h*-B_12_As_2_H_8_. Results of AIMD simulations for (**d**) *h*-B_12_N_2_H_8_, (**e**) *h*-B_12_P_2_H_8_ and (**f**) *h*-B_12_As_2_H_8_ at simulated temperatures of 300 and 600 K. The insets show the final crystal structures of *h*-B_12_X_2_H_8_ at the end of the simulation time at 600 K.

Next, we focused on examining the crystal structural stability of the hydrogenated products, which is a prerequisite for verifying the effectiveness of the hydrogenation strategy. First, we evaluated the dynamical stability by phonon spectrum calculations (Figure 2a–c), which show no imaginary frequencies for all three monolayers, indicating excellent dynamical stability. Further molecular dynamics simulations at 300 and 600 K (Figure 2d–f) demonstrate that *h*-B_12_X_2_H_8_ can maintain good thermal stability above room temperature. In addition, we also performed tests using the Born–Huang mechanical stability criteria ($C_{11}C_{22}-C_{12}^{2}>0$, and $C_{66}>0$ [41]), and the obtained elastic constants *C*_11_, *C*_22_*, C*_12_, and *C*_66_ fully satisfy the corresponding conditions, as summarized in Table 2. In summary, the three novel boron-based 2D materials obtained via the surface hydrogenation strategy possess excellent overall stability and can exist as free-standing 2D materials, representing an advantage over many borophene systems that require substrate support for stabilization.

**Figure 3:**
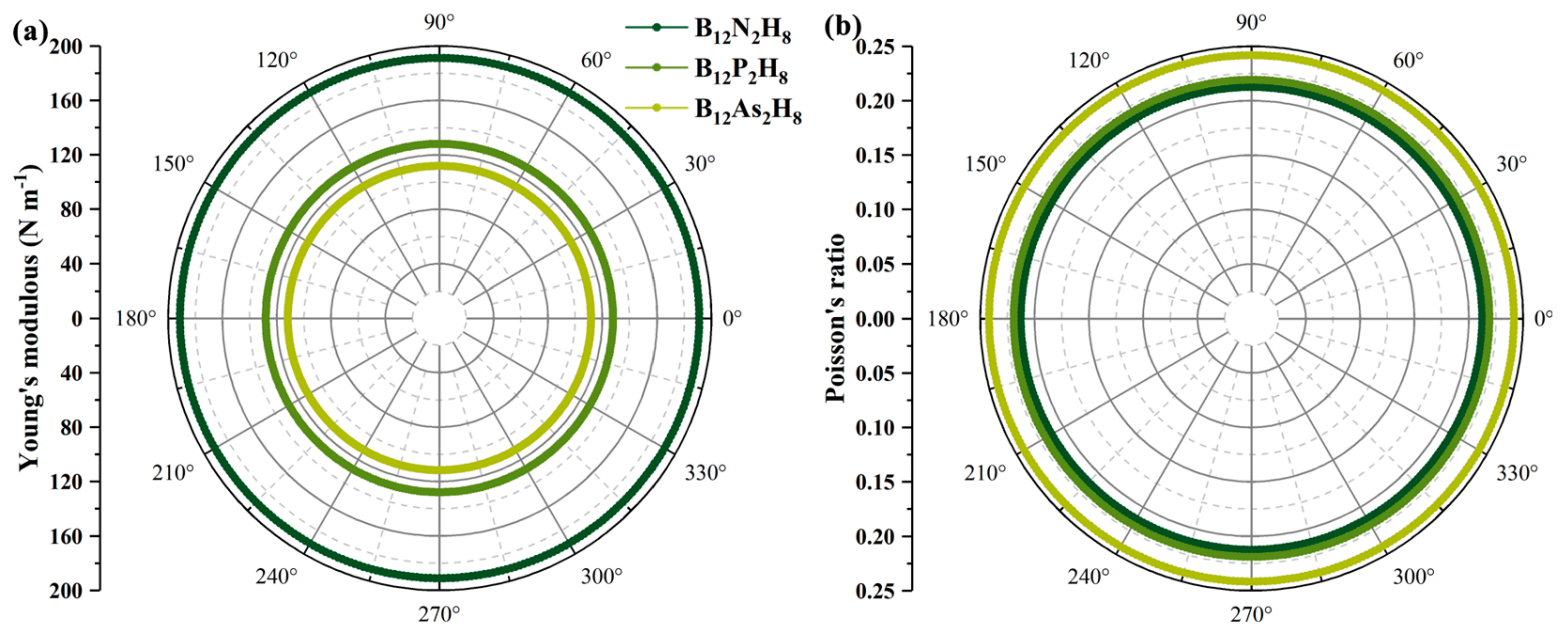
Calculated angle-dependent (**a**) Young’s modulus and (**b**) Poisson’s ratio of *h*-B_12_X_2_H_8_ (X = N, P, As).

The stability verification above lays a solid foundation for subsequent investigations into physical properties. On this basis, we systematically explored the mechanical characteristics of the *h*-B_12_X_2_H_8_ system, focusing on Young’s modulus and Poisson’s ratio, using the independent elastic constants obtained earlier. The calculation details are provided in Supplementary Material Note S1. As shown in Figure 3, both Young’s modulus and Poisson’s ratio of *h*-B_12_X_2_H_8_ exhibit typical isotropic behavior. Specifically, the Young’s moduli of *h*-B_12_N_2_H_8_, *h*-B_12_P_2_H_8_, and *h*-B_12_As_2_H_8_ are 191.10, 127.90, and 111.71 N/m, respectively, decreasing with increasing period number of the X element (Figure 3a). Young’s modulus reflects the stiffness of a material and is essentially related to bond strength. Since the electronegativities of N, P, and As decrease successively, the chemical bond strengths they form with B also gradually weaken, a trend that exactly matches the variation in Young’s modulus. Compared with the un-hydrogenated parent *h*-B_12_X_2_ phases (Table 2), the hydrogenated *h*-B_12_X_2_H_8_ exhibits a significantly increased Young’s modulus, which is also attributable to the hydrogenation-induced strengthening of bonding, further confirming the effective modification of the structural properties by hydrogenation.

Regarding Poisson’s ratio, the values for *h*-B_12_N_2_H_8_, *h*-B_12_P_2_H_8_, and *h*-B_12_As_2_H_8_ are 0.213, 0.219, and 0.242, respectively, increasing with increasing period number of X (Figure 3b), showing an opposite trend to that of Young’s modulus. Poisson’s ratio characterizes the transverse strain response of a material under applied stress; a larger value indicates a more pronounced feedback to the applied strain. Therefore, among the three systems, *h*-B_12_As_2_H_8_ exhibits the largest feedback effect, while *h*-B_12_N_2_H_8_ shows the smallest. Compared with the parent phases, the Poisson’s ratios of *h*-B_12_X_2_H_8_ are all reduced, implying that the hydrogenated materials possess higher stiffness and smaller deformation response.

When comparing *h*-B_12_X_2_H_8_ with classic two-dimensional materials: its Young’s modulus is significantly lower than that of graphene (~340 N/m) [14], but *h*-B_12_N_2_H_8_ is comparable to MoS_2_ (~200 N/m) [42] and much higher than that of black phosphorus (~40 N/m) [43]; the Poisson’s ratios are close to those of MoS_2_ (0.21) [42] and the analogous *h*-B_12_X_2_H_8_ (0.234/0.213) [44]. In summary, *h*-B_12_X_2_H_8_ can be regarded as a class of promising 2D flexible materials.

**Figure 4:**
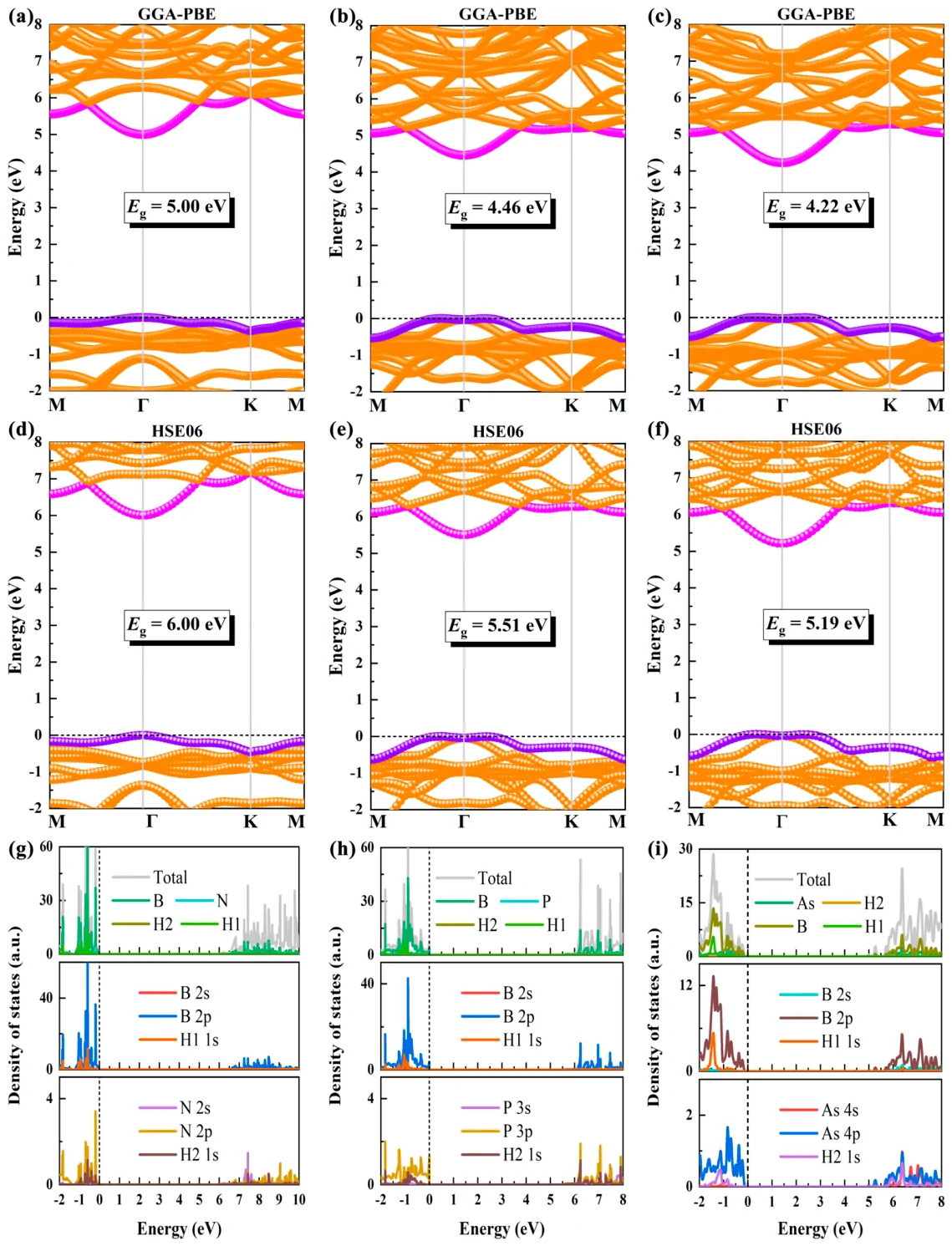
Electronic band structures of (**a**) *h*-B_12_N_2_H_8_, (**b**) *h*-B_12_P_2_H_8_ and (**c**) *h*-B_12_As_2_H_8_ calculated at GGA-PBE level. Electronic band structures of (**d**) *h*-B_12_N_2_H_8_, (**e**) *h*-B_12_P_2_H_8_ and (**f**) *h*-B_12_As_2_H_8_ calculated using hybrid functional HSE06. Density of states of (**g**) *h*-B_12_N_2_H_8_, (**h**) *h*-B_12_P_2_H_8_ and (**i**) *h*-B_12_As_2_H_8_ based on HSE06 method.

Next, we systematically analyzed the electronic structures of *h*-B_12_X_2_H_8_, and the relevant results are presented in Figure 4. First, the band structures of the three materials were calculated using the GGA-PBE method (Figure 4a–c). The calculations show that all three are wide-bandgap semiconductors (bandgap > 4 eV). Among them, *h*-B_12_N_2_H_8_ is a direct-bandgap semiconductor with a bandgap of 5.00 eV, where both the conduction band minimum (CBM) and valence band maximum (VBM) are located at the Γ point. In contrast, *h*-B_12_P_2_H_8_ and *h*-B_12_As_2_H_8_ are indirect-bandgap semiconductors with bandgaps of 4.46 eV and 4.22 eV, respectively; their VBM lies near Γ (along the Γ–M direction), while the CBM remains at Γ. As X varies from N to As, the bandgap gradually decreases, consistent with the general trend. Notably, the band structures of the latter two exhibit a “Mexican-hat” feature near Γ, which has been reported in various semiconductors [45,46].

Since GGA-PBE typically underestimates semiconductor bandgaps, we further performed band calculations using the hybrid HSE06 functional (Figure 4d–f). The band dispersion trends obtained from HSE06 are highly consistent with the PBE results, with the main difference being a significant increase in the bandgap values: the bandgaps of *h*-B_12_N_2_H_8_, *h*-B_12_P_2_H_8_, and *h*-B_12_As_2_H_8_ are raised to 6.00 eV, 5.51 eV, and 5.19 eV, respectively, corresponding to an increase of about 1 eV compared to the PBE results. Such wide bandgaps suggest that these materials hold potential for applications in high-voltage, high-temperature, and high-frequency electronic devices.

**Figure 5:**
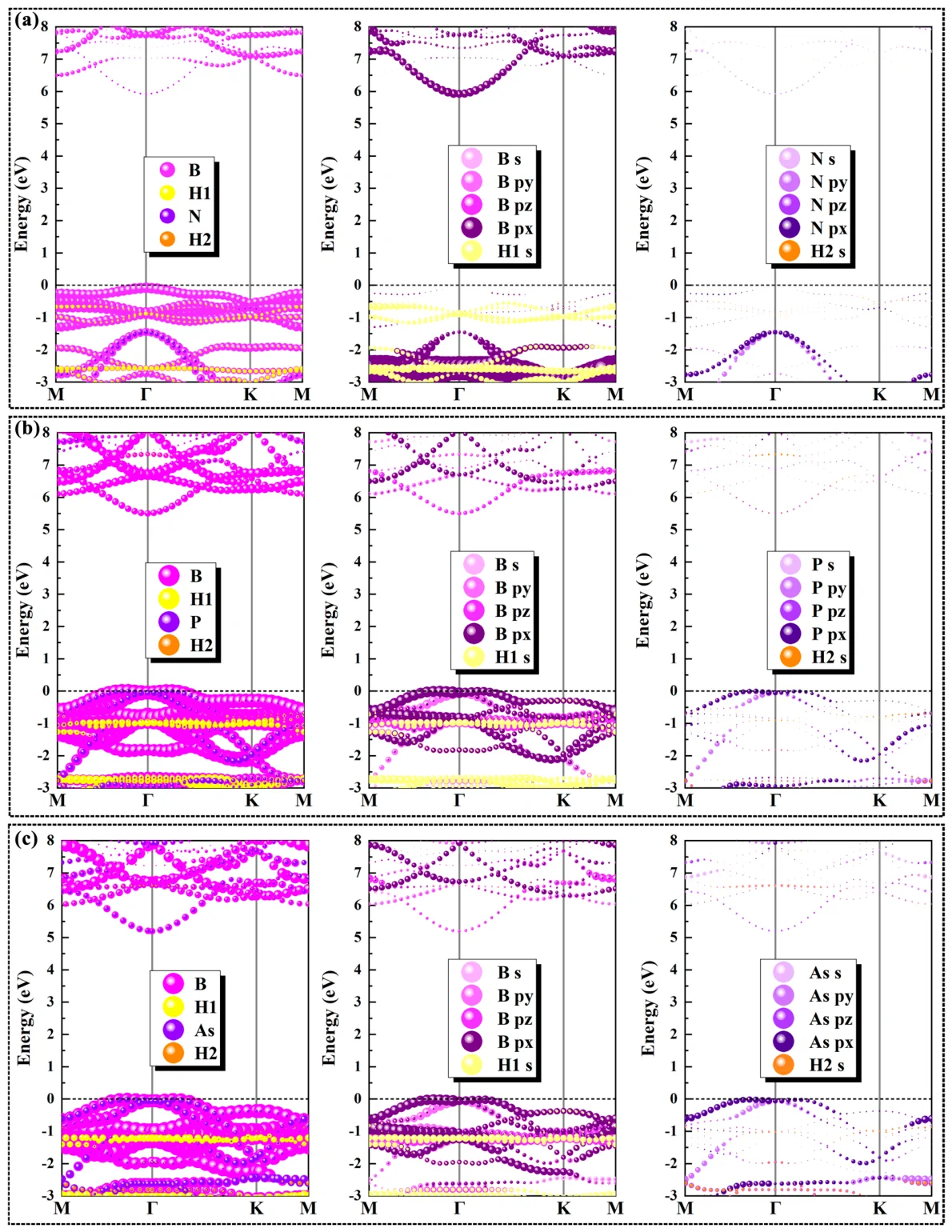
The orbital-projected band structures of (**a**) *h*-B_12_N_2_H_8_, (**b**) *h*-B_12_P_2_H_8_ and (**c**) *h*-B_12_As_2_H_8_ calculated based on hybrid functional HSE06.

Subsequently, we calculated the electronic density of states (DOS) of the three materials based on the HSE06 functional (Figure 4g–i). The results show that the electronic states mainly originate from B atoms, especially the B 2p orbitals; strong orbital hybridizations exist between B-H1 and X-H2, reflecting the effect of hydrogenation passivation. Meanwhile, the strong orbital coupling between B and X plays a key role in maintaining structural stability. To further analyze the orbital contributions, we present the orbital-projected band structures based on HSE06 (Figure 5). Overall, the valence and conduction bands of the three materials are mainly contributed by B atoms and their bonded H1 atoms, which are the dominant components of the *h*-B_12_X_2_H_8_ framework; the contributions from X atoms and their bonded H2 atoms are relatively weaker. Specific differences are as follows: in *h*-B_12_N_2_H_8_, the B p_x_ orbitals dominate both the VBM and CBM, while the H1 s and N p_x_ contributions are mainly located in the deeper valence bands. For *h*-B_12_P_2_H_8_ and *h*-B_12_As_2_H_8_, the vicinity of the valence band top is jointly dominated by the B and X p_x_ and p_y_ orbitals; the H1 s and H2 s orbitals are mainly distributed in the lower part of the valence bands, while the conduction bands are primarily contributed by the B and X p_z_ orbitals (along with minor contributions from B s and H s orbitals).

**Figure 6:**
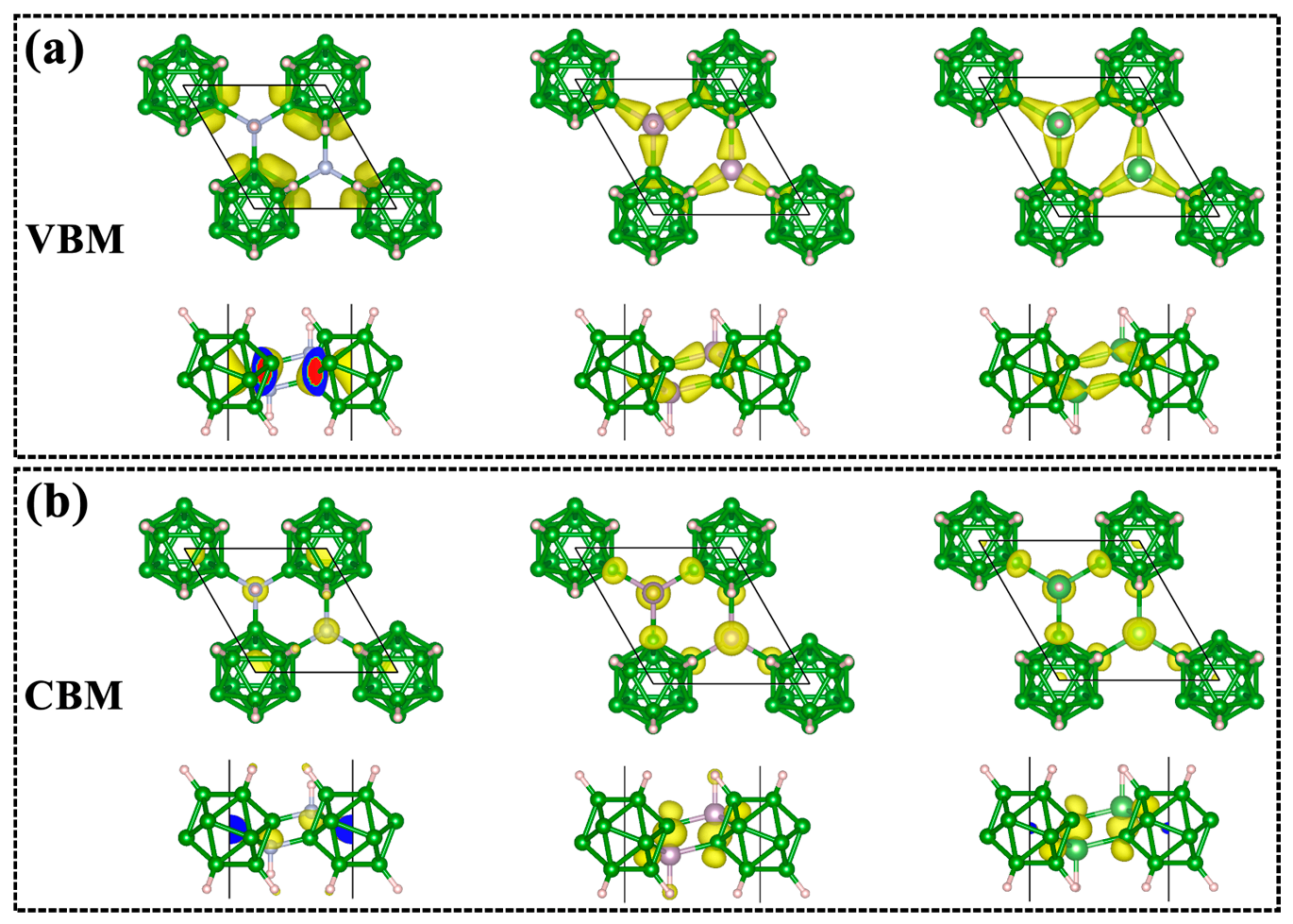
The partial charge density of (**a**) VBM and (**b**) CBM for *h*-B_12_X_2_H_8_ (X = N, P, As) calculated at GGA-PBE level. The isosurfaces are set to 0.06 *e* Å^−3^.

To more intuitively visualize the charge distributions at the VBM and CBM, we calculated the spatial charge densities based on GGA-PBE (Figure 6). 2D *h*-B_12_N_2_H_8_ exhibits pronounced differences from the other two materials: at its VBM, the charge is mainly concentrated on the B atoms bonded to N and distributed along the B–B bond direction, with almost no contribution from N atoms, which is consistent with the projected orbital analysis. In contrast, the VBM charges of *h*-B_12_P_2_H_8_ and *h*-B_12_As_2_H_8_ are primarily distributed on the B–X bonds, displaying typical sp^2^ hybridization characteristics. For the CBM, the charge in *h*-B_12_N_2_H_8_ is mainly localized on the N atoms, showing a vertical (p_z_-type) distribution, while a significant p_z_ charge density also appears at the center of the B_12_ superatom. For *h*-B_12_P_2_H_8_ and *h*-B_12_As_2_H_8_, the CBM charges mainly originate from the vertical p_z_ orbitals of P/As and the B atoms bonded to them, with minor contributions from hydrogen atoms. The above analysis indicates that the electronic properties of *h*-B_12_X_2_H_8_ generally follow a periodic trend with varying X elements, but are also influenced by the intrinsic characteristics of the elements themselves, leading to some non-monotonic variations.

Although the wide bandgap restricts the application of *h*-B_12_X_2_H_8_ in high-performance electronic devices, its carrier mobility remains a key indicator for evaluating device potential. To this end, we calculated the phonon-limited carrier mobility based on the modified deformation potential theory [47] (calculation details are provided in Supplementary Material Note S2). Table 3 lists the effective masses (*m^⁎^*) along the transport directions, the in-plane elastic moduli (*C*_2D_), and the absolute deformation potential constants (|*E*_1_|); further fitting details are provided in Figure S1. The results reveal pronounced carrier- and direction-dependent effective masses. For electrons, *h*-B_12_P_2_H_8_ and *h*-B_12_As_2_H_8_ exhibit relatively small and nearly isotropic effective masses of approximately 0.40–0.46 *m*_0_, whereas *h*-B_12_N_2_H_8_ shows a considerably larger electron effective mass of approximately 1.86 *m*_0_ along both the a and b directions. For holes, *h*-B_12_N_2_H_8_ exhibits an almost isotropic effective mass of approximately 0.54 *m*_0_. In contrast, *h*-B_12_P_2_H_8_ and *h*-B_12_As_2_H_8_ show strongly anisotropic hole effective masses, with values of approximately 2.3 *m*_0_ along the a direction and exceeding 11 *m*_0_ along the b direction, consistent with the relatively flat band dispersion along the latter direction. Moreover, the deformation potential constants obtained from band-edge fitting range from 1.939 to 6.528 eV, indicating a pronounced sensitivity of the band-edge energies to lattice strain. The mobilities calculated based on the above parameters (Table 3) show that *h*-B_12_P_2_H_8_ exhibits a relatively high hole mobility of 406–487 cm^2^V^−1^s^−1^ but a low electron mobility of 49–60 cm^2^V^−1^s^−1^. In contrast, *h*-B_12_P_2_H_8_ and *h*-B_12_As_2_H_8_ exhibit high electron mobilities of 1266–1755 and 597–688 cm^2^V^−1^s^−1^, respectively, together with much lower hole mobilities of 5–21 and 5–24 cm^2^V^−1^s^−1^, respectively. Overall, the *h*-B_12_X_2_H_8_ systems exhibit pronounced carrier-type-dependent transport behavior, with carrier mobilities spanning from a few to more than 10^3^ cm^2^V^−1^s^−1^ depending on the composition and carrier type. The values are comparable to the widely studied MoS_2_ (~200 cm^2^V^−1^s^−1^) [48], but far lower than those of ultra-high mobility materials such as black phosphorus (>10^4^ cm^2^V^−1^s^−1^) [49].

**Figure 7:**
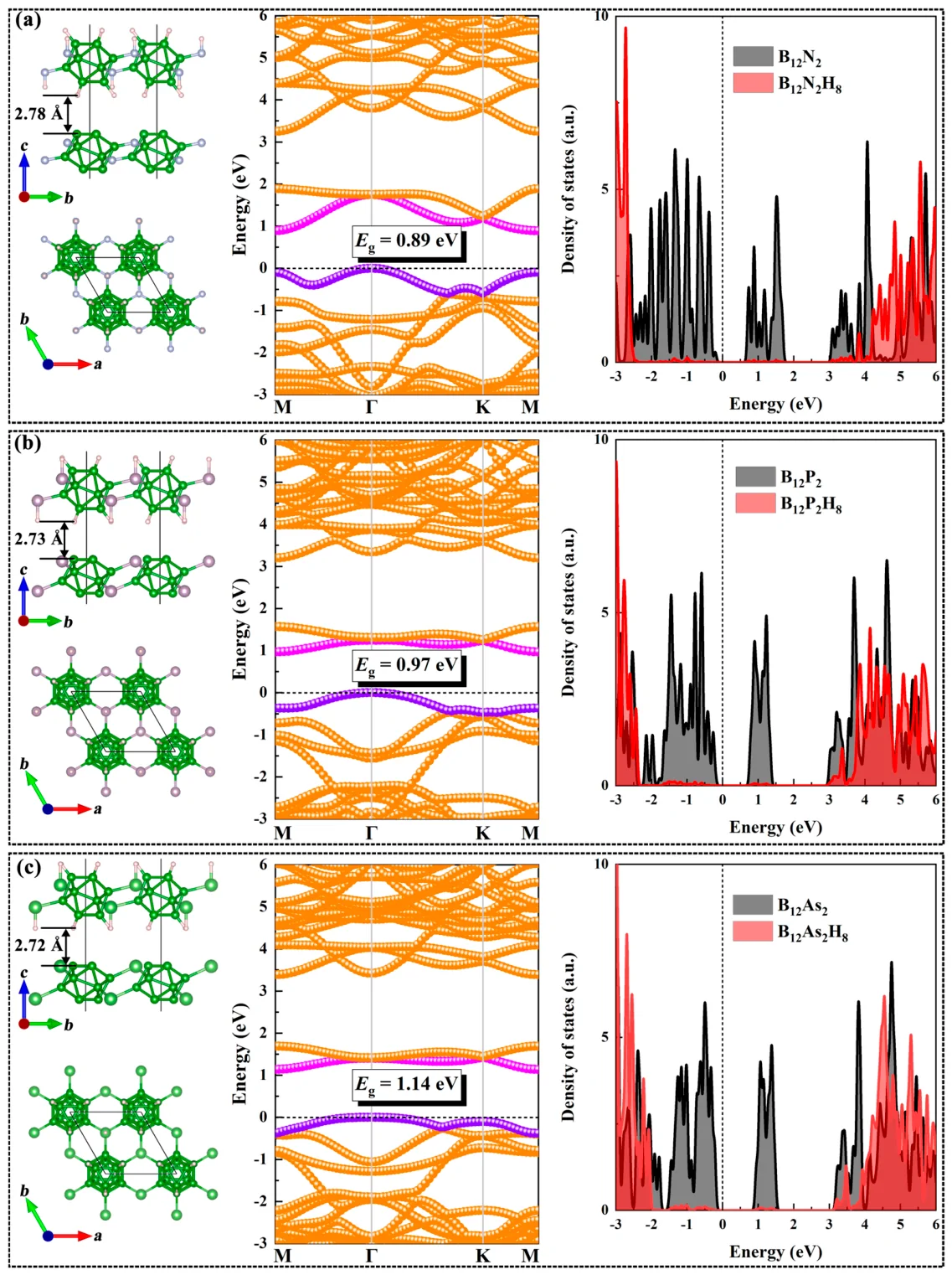
Calculated (**a**) atomic structure, band structure, and DOS for *h*-B_12_N_2_/*h*-B_12_N_2_H_8_; (**b**) *h*-B_12_P_2_/*h*-B_12_P_2_H_8_; and (**c**) *h*-B_12_As_2_/*h*-B_12_As_2_H_8_ heterojunction. All band and DOS data are from HSE06 hybrid functional calculations.

In the final part of this work, we investigated the heterojunction properties formed by the hydrogenated phase *h*-B_12_X_2_H_8_ and its parent phase *h*-B_12_X_2_, aiming to evaluate the feasibility of using *h*-B_12_X_2_H_8_ as a protective layer for *h*-B_12_X_2_ in practical applications. According to the lattice constant calculations mentioned above, the lattice mismatches between *h*-B_12_X_2_H_8_ and the corresponding parent phases are only 0.7% (*h*-B_12_As_2_H_8_/*h*-B_12_As_2_), 1.1% (*h*-B_12_P_2_H_8_/*h*-B_12_P_2_), and 1.4% (*h*-B_12_N_2_H_8_/*h*-B_12_N_2_), indicating good structural compatibility between the two. To this end, we constructed heterojunction models of *h*-B_12_X_2_H_8_/*h*-B_12_X_2_ with a 1:1 unit cell ratio (Figure 7). After structural optimization, all three heterojunctions, *h*-B_12_N_2_H_8_/*h*-B_12_N_2_, *h*-B_12_P_2_H_8_/*h*-B_12_P_2_, and *h*-B_12_As_2_H_8_/*h*-B_12_As_2_, maintained intact layered structures with interlayer spacings of 2.78, 2.73, and 2.72 Å, respectively. The calculated binding energies are –0.196 (*h*-B_12_N_2_H_8_/*h*-B_12_N_2_), –0.274 (*h*-B_12_P_2_H_8_/*h*-B_12_P_2_), and –0.319 eV/atom (*h*-B_12_As_2_H_8_/*h*-B_12_As_2_), all negative, indicating that the heterojunction formation is an exothermic and energetically favorable process.

Subsequently, we calculated the band structures and layer-projected density of states of the three heterojunctions using the hybrid functional HSE06 (Figure 7a–c). The band structure results show that all heterojunctions exhibit semiconducting characteristics with bandgaps of 0.89 (*h*-B_12_N_2_H_8_/*h*-B_12_N_2_), 0.97 (*h*-B_12_P_2_H_8_/*h*-B_12_P_2_), and 1.14 eV (*h*-B_12_As_2_H_8_/*h*-B_12_As_2_), which are in close agreement with the intrinsic bandgaps of monolayer *h*-B_12_X_2_ (0.995, 0.977, and 1.128 eV, respectively). Meanwhile, the band shapes of the heterojunctions are nearly identical to those of the parent *h*-B_12_X_2_ phases. The layer-projected density of states analysis further reveals that the band edges of *h*-B_12_X_2_H_8_ and *h*-B_12_X_2_ constitute a typical type-I band alignment, thereby almost perfectly preserving the band structure of the parent *h*-B_12_X_2_. These results indicate that *h*-B_12_X_2_H_8_ can serve as an effective protective layer for *h*-B_12_X_2_, preventing structural degradation and electronic structure alterations that may arise from contact with reactive gases in air.

## 4. Conclusions

In this study, starting from the icosahedral B_12_ superatom-based 2D materials *h*-B_12_X_2_ (X = N, P, As) as parent phases and combining surface hydrogenation with first-principles calculations, we have successfully designed a new class of highly stable boron-based 2D materials, *h*-B_12_X_2_H_8_. Our results demonstrate that hydrogenation can significantly modulate the electronic structure of the parent phases, widening the bandgap from a narrow gap of about 1 eV to a wide gap of 5.19–6.00 eV. Meanwhile, hydrogenation strengthens the bonding within the system, endowing the materials with higher Young’s modulus and lower Poisson’s ratio, thus reflecting excellent mechanical properties. Regarding carrier transport, *h*-B_12_X_2_H_8_ exhibits distinct carrier-type selectivity: *h*-B_12_N_2_H_8_ possesses a relatively high hole mobility (406–487 cm^2^V^−1^s^−1^), whereas *h*-B_12_P_2_H_8_ and *h*-B_12_As_2_H_8_ display high electron mobilities (1266–1755 and 597–688 cm^2^V^−1^s^−1^, respectively) and extremely low hole mobilities (only 5–25 cm^2^V^−1^s^−1^). More critically, when the hydrogenated phase forms a heterojunction with the parent phase, the former can serve as an ideal self-passivating protective layer, preserving the band structure of the parent phase almost intact and effectively shielding its electronic properties from external environmental perturbations, thereby offering a viable route for the practical application of *h*-B_12_X_2_ under complex working conditions. This work not only demonstrates the powerful tunability of surface hydrogenation in the functional design of B_12_ superatom-based materials, but also provides a valuable example for constructing self-passivating protective layers on 2D materials. This strategy is expected to be further extended to other superatom-cluster-based 2D systems, promoting the rational design and practical application of novel functional materials.

## Figures and Tables

**Table 1:** Calculated lattice constants *a/b* (Å), bond lengths *l* (Å), and bandgaps (in eV at the GGA-PBE and HSE06 levels) of *h*-B_12_X_2_H_8_ (X = N, P, As). The parameters of *h*-B_12_X_2_ reported in the literature are also listed in the table for reference and comparison.

**Materials**	*a*/*b*	*l*_B-B_	*l*_B-X_	*l*_B-H1_	*l*_X-H2_	$E_{g}^{\mathrm{PBE}}$	$E_{g}^{\mathrm{HS}E}$
*h*-B_12_N_2_H_8_	5.500	1.775~1.781	1.562	1.194	1.027	5.00	6.00
*h*-B_12_P_2_H_8_	6.111	1.778~1.789	1.936	1.195	1.413	4.46	5.51
*h*-B_12_As_2_H_8_	6.291	1.765~1.788	2.045	1.196	1.514	4.22	5.19
*h*-B_12_N_2_ [24]	5.580	1.597~1.912	1.468	--	--	0.057	0.995
*h*-B_12_P_2_ [24]	6.182	1.595~1.893	1.938	--	--	0.136	0.977
*h*-B_12_As_2_ [24]	6.336	1.596~1.885	2.069	--	--	0.276	1.128

**Table 2:** Calculated independent elastic constants *C*_11_/*C*_22_/*C*_12_/*C*_66_ (N/m), Young’s modulus *Y* (N/m) and Poisson’s ratio v of *h*-B_12_X_2_H_8_ (X = N, P, As). The parameters of *h*-B_12_X_2_ reported in the literature are also listed in the table for reference and comparison.

Materials	*C*_11_/*C*_22_	*C*_12_	*C*_66_	*Y*	*v*
*h*-B_12_N_2_H_8_	200.14	42.54	78.80	191.10	0.213
*h*-B_12_P_2_H_8_	134.35	29.42	52.46	127.90	0.219
*h*-B_12_As_2_H_8_	118.64	28.68	44.98	111.71	0.242
*h*-B_12_N_2_ [24]	174.84	47.79	63.53	161.77	0.273
*h*-B_12_P_2_ [24]	105.63	32.22	36.70	95.80	0.305
*h*-B_12_As_2_ [24]	90.30	28.79	30.75	81.12	0.319

**Table 3:** Calculated effective masses m* (*m*_0_) of electrons (*e*) and holes (*h*), in-plane elastic modulus *C*_2D_ (N m^−1^), deformation potential constants |*E*_1_| (eV), and corresponding carrier mobilities *μ* (cm^2^V^−1^s^−1^) for *h*-B_12_X_2_H_8_. The carrier mobility results are calculated based on the methods proposed by Lang (left, underlined) and Bardeen-Shockley (right).

**Materials**	**Carrier** **Type**	$m_{a}^{*}$	$m_{b}^{*}$	|*E*_1*a*_|	|*E*_1*b*_|	$C_{a}^{2D}$	$C_{b}^{2D}$	$\mu_{a}^{2D}$	$\mu_{b}^{2D}$
*h*-B_12_N_2_H_8_	*e*	1.866	1.860	3.602	5.553	187.52	187.81	60.48/88.55	49.15/37.43
	*h*	0.542	0.542	4.496	6.528	187.52	187.81	487.71/672.56	406.32/319.52
*h*-B_12_P_2_H_8_	*e*	0.461	0.460	3.869	1.939	127.61	127.73	1266.28/855.25	1755.85/3415.75
	*h*	2.350	11.252	3.674	2.596	127.61	127.73	20.91/16.66	5.18/6.98
*h*-B_12_As_2_H_8_	*e*	0.401	0.401	5.450	4.100	110.55	111.13	597.62/492.96	688.85/875.61
	*h*	2.321	11.399	2.906	2.724	110.55	111.13	24.53/23.35	5.17/5.44

## Data Availability

The original contributions presented in this study are included in the article and Supplementary Materials. Further inquiries can be directed to the corresponding author.

## Supplementary Materials

The following supporting information can be downloaded at: https://www.mdpi.com/article/10.3390/nano16181127/s1, Note S1: Formulas for Young’s modulus and Poisson’s ratio; Note S2: Details for carrier mobility calculation; Figure S1: The orthogonal crystal structure, band structure and fitting curves of the in-plane elastic modulus *C*_2D_ and the deformation potential constant *E*_1_ of *h*-B_12_X_2_H_8_ along a and b directions. References [45,47,50,51] are cited in the supplementary materials.
