# Supplementary material for: Surface Hydrogenation Strategy for Constructing a 2D B12X2H8 (X = N, P, As) Family with Embedded Aromatic Icosahedral B12 Superatoms
Source: Nanomaterials (Basel). 2026 Sep 9;16(18):1127. doi: 10.3390/nano16181127 (PMC13610480; doi:10.3390/nano16181127)
Supplement: Supplementary file 1 [file nanomaterials-16-01127-s001.zip › nanomaterials-4530519-supplementary.pdf]

Supporting Information for

Surface Hydrogenation Strategy for Constructing a 2D $B_{12}X_2H_8$ (X = N, P, As) Family with Embedded Aromatic Icosahedral B_{12} Superatoms

Lu-Yao Tian,[1,2] Hao-Ning Li,[3] Jun-Hui Yuan,[3,*] Pan Zhang,[1] and Jiafu Wang,[3]

[1]School of Integrated Circuits, Peking University, Beijing 100871, China

[2]School of Biomedical Engineering, South-Central Minzu University, Wuhan 430074, China

[3]School of Physics and Mechanics, Wuhan University of Technology, Wuhan 430070, Chinas

[*]Corresponding Author

E-mail: yuanjh90@163.com (J.-H. Yuan)

Note S1. Formulas for Young's modulus *Y* and Poisson's ratio *v*.

For the angle dependent in-plane Young's modulus (Y) and Poisson's ratio (υ) are calculated using the independent elastic constants (C_{11}, C_{22}, C_{12} and C_{66}). The calculation formula is as follows [1]:

$$Y(\theta) = \frac{C_{11}C_{22} - C_{12}^2}{C_{11}sin^4\theta + C_{22}cos^4\theta + \left(\frac{C_{11}C_{22} - C_{12}^2}{C_{66}} - 2C_{12}\right)cos^2\theta sin^2\theta}$$

$$\nu(\theta) = \frac{C_{12}(sin^4\theta + cos^4\theta) - (C_{11} + C_{22} - \frac{C_{11}C_{22} - C_{12}^2}{C_{66}})cos^2\theta sin^2\theta}{C_{11}sin^4\theta + C_{22}cos^4\theta + \left(\frac{C_{11}C_{22} - C_{12}^2}{C_{66}} - 2C_{12}\right)cos^2\theta sin^2\theta}$$

Note S2. Details for carrier mobility calculation.

The acoustic-phonon limited mobility can be obtained initially based on the deformation potential theory proposed by Bardeen and Shockley (BS for short)[2]. That is, if treating the semiconductor as a homogeneous media, the carrier mobility of 2D materials can be calculated by the following equation:

$$\mu_{2D} = \frac{e\hbar^3 C_{2D}}{k_B T m^* m_d (E_l^i)^2},$$

where $\hbar$ is the reduced Planck constant, k_B is the Boltzmann constant, m^* is the effective mass in the transport direction, m_d is the average effective mass determined by $m_d = (m_a^* m_b^*)^{1/2}$, and T is the temperature (we use T = 300 K). The elastic modulus C_{2D} of the longitudinal strain in the propagation direction is derived from $(E - E_0)/S_0 = C_{2D}(\Delta l/l_0)^2/2$, where E is the total energy of the 2D structure, and S_0 is the lattice area of the equilibrium supercell. The deformation potential constant E_l^i is defined as $E_l^i = \Delta E_i/(\Delta l/l_0)$. Here ΔE_i is the energy change of the ith band under proper cell compression and dilatation (calculated using a step of 0.5%), l_0 is the lattice constant in the transport direction and Δl is the deformation of l_0. Such formula may be used when the elastic constants and the deformation potential constants are

relatively homogenous over the *a*-*b* plane for 2D semiconductors.

Nevertheless, as the deformation potential constants show great anisotropy, the above ultra-high mobilities are questionable. For instance, an electron experiences less scattering from the acoustic phonons from the *b*-direction, but the phonons from *a*-direction may impose strong scattering on the same electron. In a recent work by Lang *et al.*[3], a new formula for the acoustic phonon-limited mobility in 2D semiconductors was given, taking into the account of the anisotropic elastic constants or deformation potential constants:

$$\mu_{\mathrm{a}} = \frac{e\hbar^3}{k_{\mathrm{B}}T(m_a^*)^{3/2}(m_b^*)^{1/2}}\left(\frac{A+B-\sqrt{A^2-B^2}}{B\sqrt{A^2-B^2}}\right)\left(\frac{I+J-\sqrt{I^2-J^2}}{J\sqrt{I^2-J^2}}\right),$$

where the parameters A, B, I, J are defined as

$$A = \overline{E_l}^2 + \frac{\Delta E_l^2}{2}, \quad B = E_l \Delta E_l$$

$$I = \frac{1}{\sqrt{\overline{C}^2 - \Delta C^2}}, \quad J = \frac{\overline{C}}{\Delta C}\left(\frac{1}{\overline{C}} - I\right)$$

Here the terms representing averages and differences are

$$\overline{E_l} = \frac{E_{lb} + E_{la}}{2}, \quad \Delta E_l = \frac{E_{lb} - E_{la}}{2}$$

$$\overline{C} = \frac{C_{22} + C_{11}}{2}, \quad \Delta C = \frac{C_{22} - C_{11}}{2}$$

Such formula successfully eliminates the artificial strong anisotropy in several 2D semiconductors[1,4].

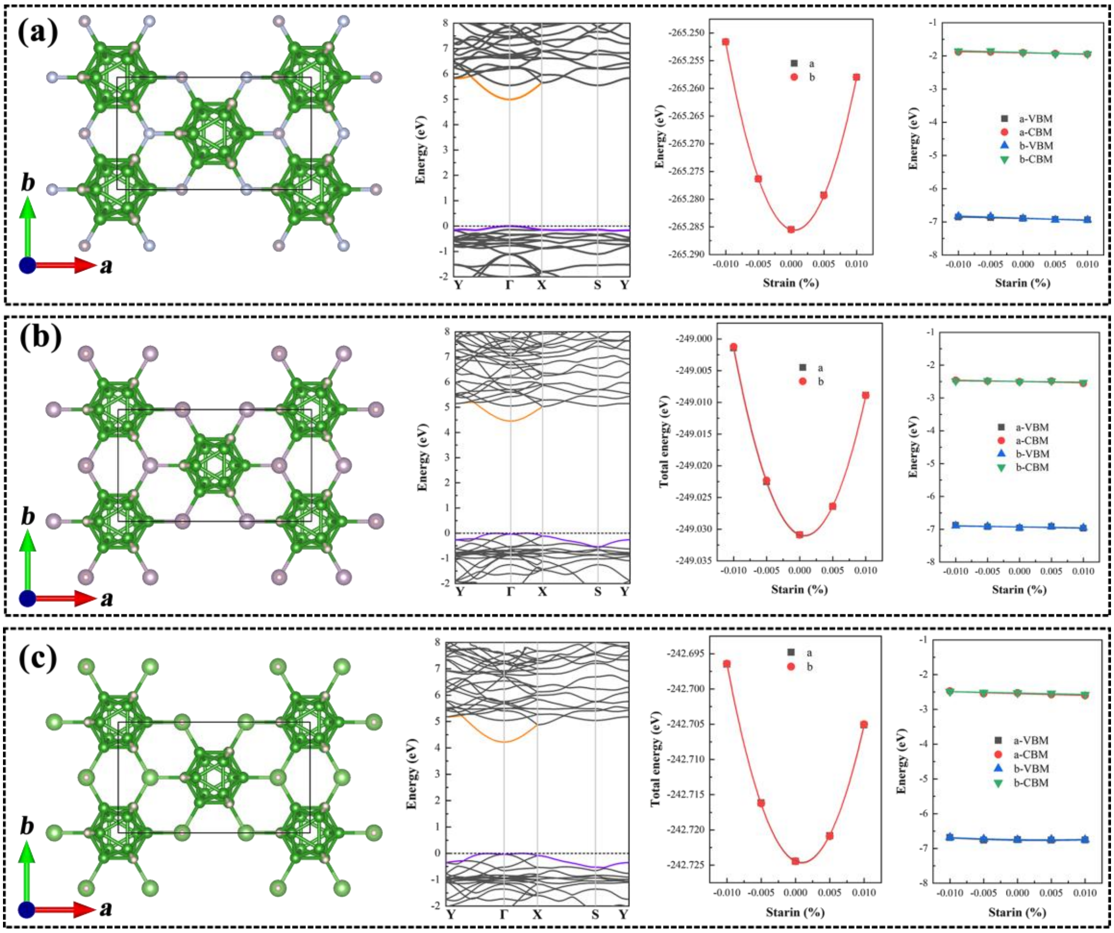

Figure S1. The orthogonal crystal structure, band structure and fitting curves of the in-plane elastic modulus C_{2D} and the deformation potential constant E_1 of (a) h-$B_{12}N_2H_8$, (b) h-$B_{12}P_2H_8$ and (c) h-$B_{12}As_2H_8$ along *a* and *b* directions, respectively.

References

1. Yuan, J.-H.; Xue, K.-H.; Wang, J.-F.; Miao, X.-S. Gallium Thiophosphate: An Emerging Bidirectional Auxetic Two-Dimensional Crystal with Wide Direct Band Gap. *J. Phys. Chem. Lett.* **2019**, *10*, 4455–4462, doi:10.1021/acs.jpclett.9b01611.
2. Bardeen, J.; Shockley, W. Deformation Potentials and Mobilities in Non-Polar Crystals. *Phys. Rev.* **1950**, *80*, 72–80, doi:10.1103/PhysRev.80.72.
3. Lang, H.; Zhang, S.; Liu, Z. Mobility Anisotropy of Two-Dimensional Semiconductors. *Phys. Rev. B* **2016**, *94*, 235306, doi:10.1103/PhysRevB.94.235306.
4. Song, Y.-Q.; Yuan, J.-H.; Li, L.-H.; Xu, M.; Wang, J.-F.; Xue, K.-H.; Miao, X.-S. KTlO: A Metal Shrouded 2D Semiconductor with High Carrier Mobility and Tunable Magnetism. *Nanoscale* **2019**, *11*, 1131–1139, doi:10.1039/C8NR08046A.
